# Application of Homochiral Alkylated Organic Cages as Chiral Stationary Phases for Molecular Separations by Capillary Gas Chromatography

**DOI:** 10.3390/molecules21111466

**Published:** 2016-11-08

**Authors:** Shengming Xie, Junhui Zhang, Nan Fu, Bangjin Wang, Cong Hu, Liming Yuan

**Affiliations:** Department of Chemistry, Yunnan Normal University, Kunming 650500, China; xieshengming_2006@163.com (S.X.); zjh19861202@126.com (J.Z.); funan123@163.com (N.F.); wangbangjin711@163.com (B.W.); hucong657312@163.com (C.H.)

**Keywords:** porous organic cage, capillary column, chiral stationary phase, chiral separation, gas chromatography

## Abstract

Molecular organic cage compounds have attracted considerable attention due to their potential applications in gas storage, catalysis, chemical sensing, molecular separations, etc. In this study, a homochiral pentyl cage compound was synthesized from a condensation reaction of (*S*,*S*)-1,2-pentyl-1,2-diaminoethane and 1,3,5-triformylbenzene. The imine-linked pentyl cage diluted with a polysiloxane (OV-1701) was explored as a novel stationary phase for high-resolution gas chromatographic separation of organic compounds. Some positional isomers were baseline separated on the pentyl cage-coated capillary column. In particular, various types of enantiomers including chiral alcohols, esters, ethers and epoxides can be resolved without derivatization on the pentyl cage-coated capillary column. The reproducibility of the pentyl cage-coated capillary column for separation was investigated using nitrochlorobenzene and styrene oxide as analytes. The results indicate that the column has good stability and separation reproducibility after being repeatedly used. This work demonstrates that molecular organic cage compounds could become a novel class of chiral separation media in the near future.

## 1. Introduction

The separation of chiral compounds is one of the most interesting and challenging tasks in the field of separation science [1], because enantiomers show identical chemical and physical properties in an achiral environment. Chromatographic techniques such as high performance liquid chromatography (HPLC), gas chromatography (GC), supercritical fluid chromatography (SFC), thin layer chromatography (TLC), and capillary electrochromatography (CEC) are still the most convenient and cost-effective approaches to obtain optically pure compounds [2]. Among them, HPLC and capillary GC are the most reliable and commonly employed analytical techniques for the separation of enantiomers [3,4]. Compared to other chromatographic techniques, capillary GC possesses the advantages of high-resolution, high-efficiency, sensitivity, fast analysis and absence of liquid mobile phases. Therefore, it is very necessary to continue developing novel chiral materials as stationary phases with high resolution and excellent enantioselectivity capable of separating a wide variety of chiral compounds in GC.

In recent years, porous materials containing some unusual properties such as diverse compositions and structures, high surface areas, ordered porosity, good chemical stability, tunable pore size and so on, have attracted significant attention in many areas, including gas adsorption and storage, catalysis, separation, etc. In general, porous materials can be designed and synthesized by using two main synthetic strategies: extended networks and discrete organic cage molecules. To date, there are various kinds of chiral porous network materials, such as metal-organic frameworks (MOFs) [5,6,7,8,9,10,11,12,13,14], porous organic frameworks (POFs) [15,16] and inorganic mesoporous materials [17,18], which have been used as chiral chromatographic stationary phases for separation of enantiomers. Unlike network materials, porous organic cages (POCs) are composed of discrete molecules with intermolecular forces rather than covalent or coordination bonds [19]. In past few years, many shape-persistent POCs have been designed and explored for various areas (e.g., gas adsorption, molecular separation, heterogeneous catalysis, and sensing) by several research groups [20,21,22,23,24]. In 2008, an imine-linked [4+6] tetrahedral cage has been synthesized through imine condensation of 1,3,5-triformylbenzene and (*R*,*R*)-1,2-cyclohexanediamine by Gawronski et al. [25], but the crystal structure of the tetrahedral cage was not studied. Subsequently, Cooper and co-workers reported a series of new POCs obtained through the condensation of 1,3,5-triformylbenzene or tris(4-formylphenyl)amine with various diamines [26,27,28,29]. Surprisingly, these cage molecules can self-assemble into crystalline materials with permanent porosity through weak intermolecular forces and separate rare gases and some organic molecules [30,31]. An important advantage of POCs is their good solubility in common organic solvents, which makes them suitable for use as separation media in capillary GC. In February 2015, our group first proved useful commercially of a homochiral POC in GC enantioseparations and applied China’s patent [32]. Subsequently, the application of POCs in GC chiral separation has been further developed. Recently, several homochiral POCs (e.g., CC3-R [33,34], CC9 [35] and CC10 [36]) useful as capillary GC stationary phases for enantioselective separation of enantiomers have been reported by the Cooper group and our group. These POCs-based stationary phases exhibited excellent chiral recognition ability toward a wide range of chiral compounds. 

Cooper and co-workers reported the syntheses of homochiral alkylated organic cages functionalised with twelve *n*-hexyl, *n*-pentyl, isohexyl or *n*-octyl tails by a one-step [6+4] condensation of the corresponding (*S*,*S*)-1,2-functionalised-1,2-diamines with 1,3,5-triformylbenzene in chloroform for 72 h at 60 or 65 °C using trifluoroacetic acid as the catalyst [37]. Herein we report the use of a homochiral pentyl cage diluted with polysiloxane (OV-1701) as chiral stationary phase for high-resolution capillary GC separation. The pentyl cage-coated capillary column exhibits good selectivity and chiral recognition ability towards a wide variety of racemates belonging to different classes such as chiral alcohols, esters, ethers and epoxides, especially for chiral alcohols. Besides, the pentyl cage-based capillary column also offers good performance for the separation of positional isomers.

## 2. Results and Discussion

### 2.1. Characterization of the Synthesized Pentyl Cage and the Pentyl Cage-Coated Capillary Column

**Figure 1:**
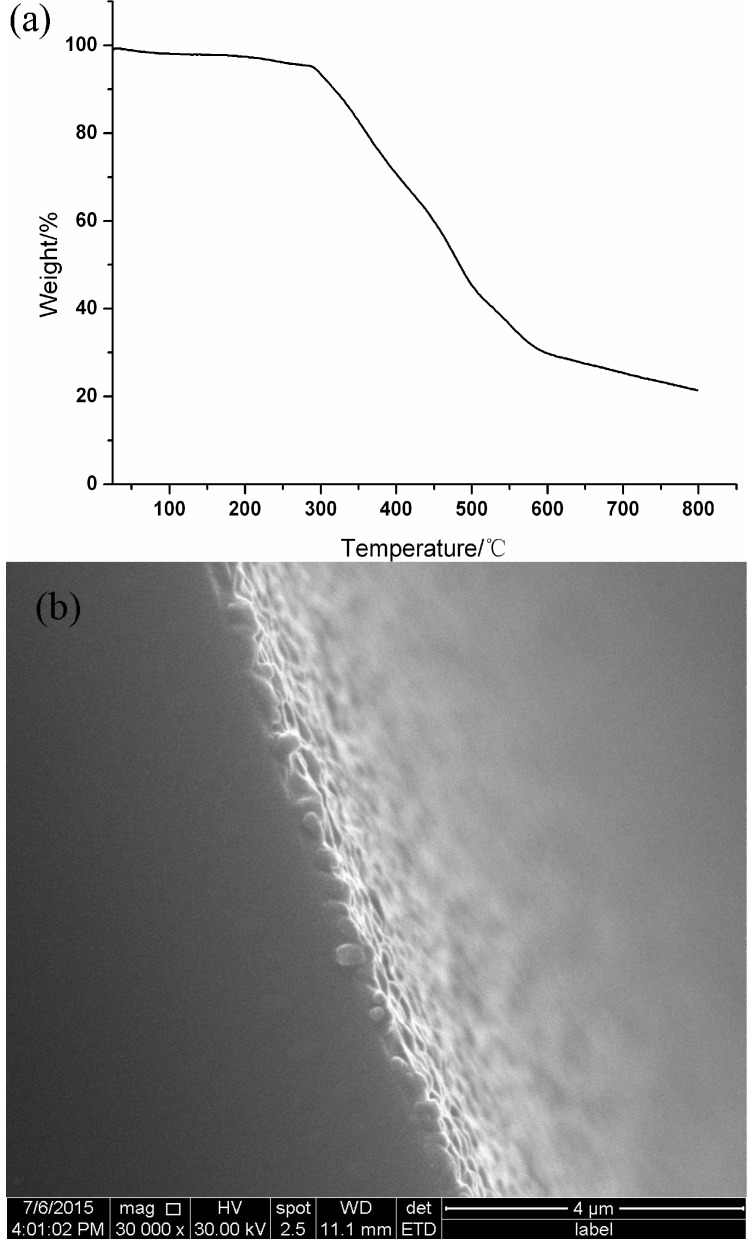
(**a**) TGA of pentyl cage; (**b**) SEM image of the thickness of stationary phase coating in part of the pentyl cage-coated capillary column.

NMR data analysis (Figures S1 and S2, Supplementary Materials) demonstrates that the pentyl cage was successfully synthesized. The corresponding TGA curve reveals that the pentyl cage is at least stable up to 290 °C, indicating its suitability for use in GC (Figure 1a). The inner surface of the pentyl cage-coated capillary column was characterized by SEM. Figure 1b shows the SEM image of the inner wall on the fabricated column. As can be seen from SEM image, a thin and uniform coating with about 260 nm thickness was formed on the inner wall of the capillary column.

Column efficiency of the pentyl cage-coated capillary column was measured by using *n*-dodecane as analyte at 120 °C. The number of theoretical plates of the capillary column was 3510 plates∙m^−1^, further indicating the good coating performance of the pentyl cage.

McReynolds constants are used to evaluate the polarity of a stationary phase. The polarity of pentyl cage-coated capillary column was determined using benzene, 1-butanol, 2-pentanone, 1-nitropropane, and pyridine as probe compounds (Table 1). The McReynolds constants of the five selected analytes represent various stationary phase characteristics (e.g., dispersion forces, hydrogen-bonding ability, electron donor and acceptor ability, dipolar and acidic character, etc.) interacting with the analytes. Squalane was used as a standard nonpolar stationary phase, and the McReynolds constants of pentyl cage-coated capillary column were compared to those of squalane. The average of the five McReynolds constants is 130.6, revealing a moderate polarity of the pentyl cage-coated capillary column.

### 2.2. Separation of Positional Isomers on the Pentyl Cage-Coated Capillary Column

**Figure 2:**
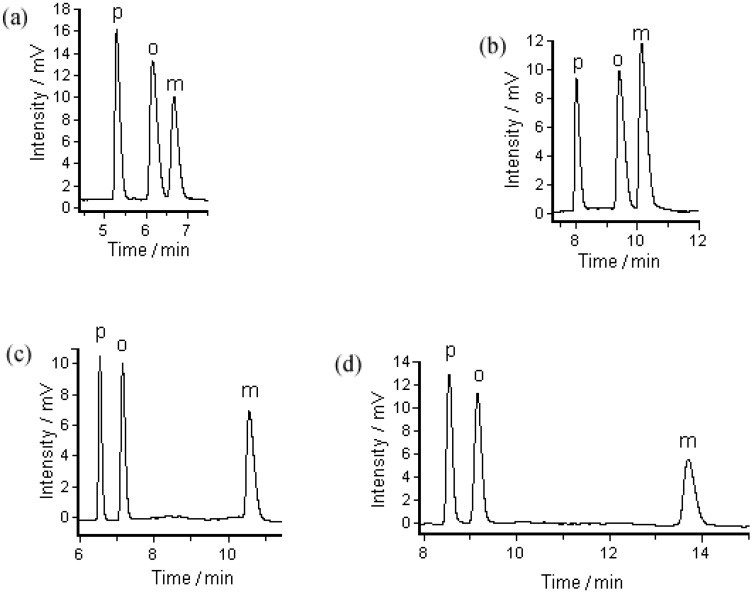
GC chromatograms using the pentyl cage-coated capillary column for separation of positional isomers: (**a**) *o*-, *m*-, and *p*-dibromobenzene under a N_2_ flow rate of 13.8 cm∙s^−1^ at 90 °C; (**b**) *o*-, *m*-, and *p*-dibromobenzene under a N_2_ flow rate of 13.8 cm∙s^−1^ at 110 °C; (**c**) *o*-, *m*-, and *p*-nitrochlorobenzene under a N_2_ flow rate of 14.9 cm∙s^−1^ at 115 °C; (**d**) *o*-, *m*-, and *p*-nitrobromobenzene under a N_2_ flow rate of 14.9 cm∙s^−1^ at 120 °C.

High-resolution separation of positional isomers is of significant importance in the chemical industry and environmental analysis [38]. However, it is a challenging task to separate some positional isomers owing to their similar physical and chemical properties. So far, capillary GC is one of the most efficient methods for the separation of various positional isomers. To investigate the separation properties of the pentyl cage-coated capillary column, some positional isomers were selected as test solutes. The pentyl cage-coated capillary column offered good separation of some isomer mixtures, including dichlorobenzene, dibromobenzene, nitrochlorobenzene, and nitrobromobenzene. Molecular structures of these isomers are shown in Figure S3 (Supplementary Materials). The chromatograms and results of separation of isomer mixtures on pentyl cage-coated capillary column are shown in Figure 2 and Table 2, respectively. As can be seen from Figure 2, baseline separation of all isomers were achieved on the pentyl cage-coated capillary column. Interestingly, the elution sequence of all isomers followed an increasing order of *para*-isomer < *ortho*-isomer < *meta*-isomer, rather than the order of their boiling points (e.g., *meta*-dibromobenzene (218 °C) < *para*-dibromobenzene (219 °C) < *ortho*-dibromobenzene (225 °C)). All the *meta*-isomers eluted much later than the *ortho*- and *para*-isomers on the pentyl cage-coated capillary column, indicating higher selectivity and stronger retention behavior toward *meta*-isomers than the *ortho*- and *para*-isomers. This experimental result is in agreement with previously reported data for POCs-based chiral stationary phases such as CC3-R, CC9 and CC10 when used for GC separation of positional isomers [34,35,36]. The main reason for this can probably be attributed to the *meta*-substituted aryl face geometry of the building unit (1,3,5-triformylbenzene) which was employed for the synthesis of the pentyl cage and the abovementioned POCs (CC3-R, CC9 and CC10). In other words, the molecular geometry of *meta*-substituted isomers will better match with pentyl cage molecules, resulting in longer retention times for *meta*-isomers than those of *ortho*- and *para*-isomers on the pentyl cage-coated capillary column.

### 2.3. Separation of Enantiomers on the Pentyl Cage-Coated Capillary Column

**Figure 3:**
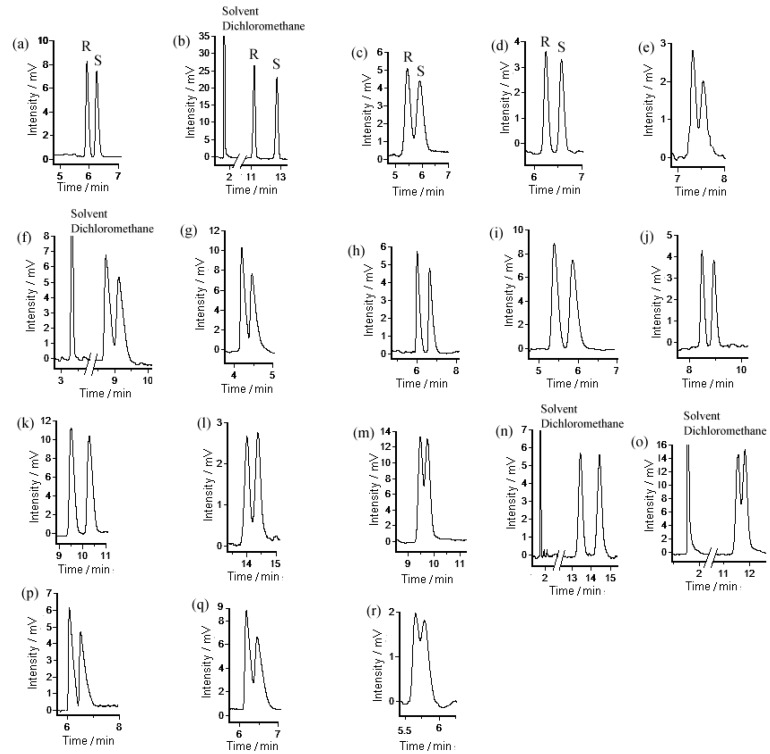
GC chromatograms on the pentyl cage-coated capillary column for separation of racemates: (**a**) styrene oxide; (**b**) trans-stilbene oxide; (**c**) 1,2-epoxybutane; (**d**) 1,2-epoxyhexane; (**e**) 1-bromo-2,3-epoxypropane; (**f**) *n*-butyl glycidyl ether; (**g**) 1-methoxy-2-hydroxypropane; (**h**) 1-methoxy-2-butanol; (**i**) 3-butyn-2-ol; (**j**) 1-phenylethanol; (**k**) 1-phenyl-1-propanol; (**l**) 4-chlorophenethylalcohol; (**m**) 2-phenyl-1-propanol; (**n**) 1-naphthylethanol; (**o**) 1-(2-naphthyl)ethanol; (**p**) methyl 3-hydroxybutyrate, (**q**) ethyl 3-hydroxybutyrate; (**r**) γ-valerolactone. The separation conditions are shown in Table 3.

As a new class of chiral materials, homochiral POCs have been attracting attention because of their potential applications in enantioselective separation [33,34,35,36]. To investigate the chiral resolution ability of the pentyl cage, a great deal of enantiomers were analyzed without derivatization on the pentyl cage-coated capillary column. We found that this column exhibited good chiral separation performance toward various types of enantiomers, including alcohols, esters, ethers and epoxides, especially for chiral alcohols. Figure S4 (Supplementary Materials) shows the molecular structures of the enantiomers. The retention factor (k_1_) for the first eluted enantiomers, separation factor (α) and resolution (Rs) are listed in Table 3. The resolution chromatograms of enantiomers are given in Figure 3. The chromatograms show baseline or at least 80% valley separation for all enantiomers except for 2-phenyl-1-propanol, 1-(2-naphthyl)ethanol, ethyl 3-hydroxybutyrate and γ-valerolactone. Notably, a high-resolution gas chromatographic enantioseparation of trans-stilbene oxide (Rs = 4.94) was achieved on the pentyl cage-coated capillary column.

The pentyl cage has a tetrahedral cage structure with twelve *n*-pentyl tails formed by imine condensation between four 1,3,5-triformylbenzene molecules and six (*S*,*S*)-1,2-pentyl-1,2-diamino-ethanes (Figure S5, Supplementary Materials). It is very difficult to completely understand the chiral recognition mechanisms for enantioseparation on a chiral stationary phase because the influence of the chiral microenvironment on the chiral properties of chromatographic systems is complicated [39]. Many different classes of enantiomers can be separated on the pentyl cage-coated capillary column. There is no doubt that only one retention mechanism cannot explain all these chiral chromatographic resolution results. Therefore, chiral recognition may depend on multimodal enantioselective retention mechanisms existing in the pentyl cage, which may involve chiral steric fits, van der Waals forces, hydrogen-bondings, dispersion forces, dipole-dipole interactions and π-π interactions, etc. For instance, the pentyl cage-coated capillary column offered good resolution for chiral alcohols, suggesting chiral discrimination mainly ascribable to a specific interaction between the hydroxyl group of chiral alcohols and the nitrogen atom in the imine of pentyl cage, and other interactions also affect the chiral recognition. In addition, other enantiomers such as esters, ethers and epoxides, which either contain no hydrogen bonding groups or contain only hydrogen bonding acceptor groups and/or have permanent dipole moments, were also resolved [34]. Consequently, dipole-dipole interactions as well as other interactions, including van der Waals forces, dispersion forces and π-π interactions between enantiomers and pentyl cage, are most responsible for chiral recognition.

### 2.4. The Reproducibility of the Pentyl Cage-Coated Capillary Column for Separation

**Figure 4:**
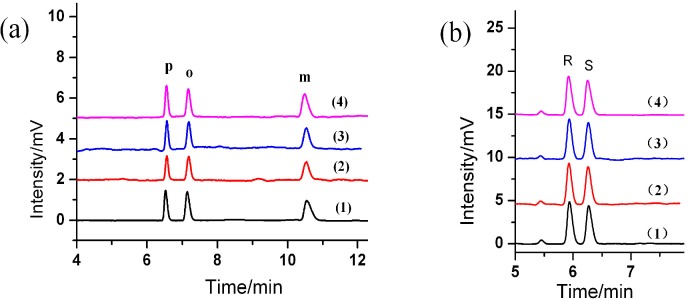
Reproducible chromatograms on the pentyl cage-coated capillary column for the separation of (**a**) *o*-, *m*-, and *p-*nitrochlorobenzene at 115 °C under a N_2_ flow rate of 14.9 cm∙s^−1^; (**b**) styrene oxide at 110 °C under a N_2_ linear velocity of 10.8 cm∙s^−1^ (1) Chromatograms obtained before the column were repeatedly used; (2), (3) and (4) chromatograms obtained after the columns have been subjected to 100, 300, and more than 500 injections, respectively.

Separation reproducibility of the pentyl cage-coated capillary column was investigated using nitrochlorobenzene and styrene oxide as examples. The reproducible chromatograms of nitrochlorobenzene and styrene oxide, separated before and after the columns have been subjected to 100, 300, and more than 500 injections (Figure 4). From Figure 4, no significant changes in retention time and recognition ability were observed, indicating good stability and reproducibility of the pentyl cage-coated capillary column for GC separation.

## 3. Experimental Section

### 3.1. Reagents and Materials

All chemicals and reagents used were at least of analytical grade. All racemates were purchased from Sigma-Aldrich (St. Louis, MO, USA), Adamas-beta (Shanghai, China) or TCI (Tokyo, Japan). The positional isomers (dichlorobenzenes, dibromobenzenes, nitrochlorobenzene, and nitrobromo-benzenes) were obtained from Aladdin Chemistry Co. Ltd. (Shanghai, China). Hexanal, (*R*,*R*)-1,2-bis(2-hydroxyphenyl)-1,2-diaminoethane and 1,3,5-triformylbenzene were acquired from Acros (Geel, Belgium) and Sigma-Aldrich. Chloroform, dichloromethane, toluene, methanol, diethyl ether and acetone were from Tianjin Fengchuan Fine Chemical Research Institute (Tianjin, China). Trifluoroacetic acid was purchased from Alfa Aesar (Shanghai, China). The untreated fused-silica capillary column (0.25 mm inner diameter) was purchased from Yongnian Chromatogram Apparatus Co. Ltd. (Yongnian County, Hebei, China).

### 3.2. Instrumentations

A Shimadzu GC-2014C system (Kyoto, Japan) equipped with a flame ionization detector (FID), split injection port and capillary control unit was employed for all GC separations. The data acquisition was performed on a N-2000 chromatography data system (Zhida Information Engineering Co. Ltd., Zhejiang University, China). High purity N_2_ (99.999%) was used as the carrier gas. ^1^H- and ^13^C-NMR spectra were recorded on a Bruker DRX 500 NMR ultrashield spectrometer (Karlsruhe, Germany). Scanning electron microscopy (SEM) images were carried out on a FEI Quanta FEG 650 scanning electron microscope (Hillsboro, OR, USA). Thermogravimetric analysis (TGA) was performed on a ZRY-1P simultaneous thermal analyzer (Shanghai, China) from room temperature to 800 °C at a ramp rate of 10 °C∙min^−1^.

### 3.3. Synthesis of (S,S)-N,N’-bis(Salicylidene)-1,2-pentyl-1,2-diaminoethane

**Scheme 1:**
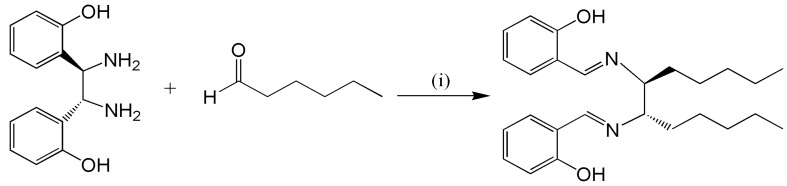
Synthesis of (*S*,*S*)-*N,N’*-bis(salicylidene)-1,2-pentyl-1,2-diaminoethane. *Reagents and Conditions*: (i) Toluene, Dean-Stark apparatus.

(*S*,*S*)-*N*,*N’*-bis(Salicylidene)-1,2-pentyl-1,2-diaminoethane was synthesized according to a previous literature report [37] (Scheme 1). Hexanal (2.46 mL, 20.50 mmol) was added to a solution of (*R*,*R*)-1,2-bis(2-hydroxyphenyl)-1,2-diaminoethane (2.0 g, 8.2 mmol) in toluene (50 mL) at room temperature. Subsequently, the resulting solution was refluxed overnight with a Dean-Stark trap. After removal of the solvent under reduced pressure, the resulting viscous yellow oil was purified by precipitation using methanol. Finally, a yellow solid (1.98 g, yield 59%) was obtained. ^1^H-NMR (CDCl_3_): δ 13.48 (br s, 2H), 8.28 (s, 2H), 7.29 (ddd, ^3^*J*_HH_ = 9 Hz, ^4^*J*_HH_ = 1 Hz 2H), 7.23 (dd, ^3^*J*_HH_ = 9 Hz, ^4^*J*_HH_ = 1 Hz, 2H), 6.98 (d, ^3^*J*_HH_ = 6 Hz, 2H), 6.85 (ddd, ^3^*J*_HH_ = 6 Hz, ^4^*J*_HH_ = 1 Hz, 2H), 3.32–3.28 (m, 2H), 1.69 (m, 4H), 1.29 (m, 12H), 0.88 (t, ^3^*J*_HH_ = 6 Hz, 6H). ^13^C-NMR (CDCl_3_): δ 164.8, 161.3, 132.2, 131.3, 118.5, 118.5, 117.1, 73.7, 32.5, 31.6, 25.9, 22.6, 14.0.

### 3.4. Synthesis of (S,S)-1,2-Pentyl-1,2-diaminoethane

**Scheme 2:**
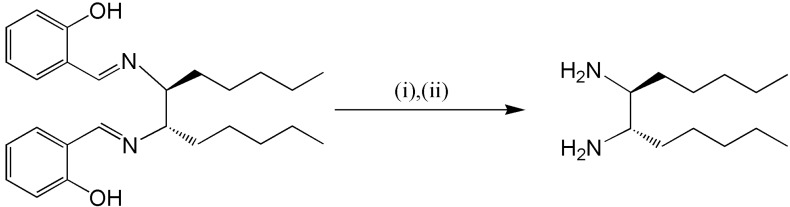
Synthesis of (*S*,*S*)-1,2-pentyl-1,2-diaminoethane. *Reagents and Conditions*: (i) THF/HCl 37% solution, ambient temperature for 24 h; (ii) NaOH 1M.

(*S*,*S*)-1,2-pentyl-1,2-diaminoethane was synthesized according to a previous literature procedure [37] (Scheme 2). (*S*,*S*)-*N*,*N’*-bis(Salicylidene)-1,2-pentyl-1,2-diaminoethane (2.6 mmol) was dissolved in 12 mL of THF, then a mixture of 0.78 mL of 37% HCl solution and 12 mL of THF was added and stirred at ambient temperature for 24 h. Subsequently, the mixture was diluted with 50 mL of diethyl ether and extracted three times with 15 mL of water. The water phase was basified using NaOH 1.0 M, extracted three times with 30 mL of dichloromethane and dried over dry Na_2_SO_4_. (*S*,*S*)-1,2-Pentyl-1,2-diaminoethane (0.31g, yield 57.9%) was obtained as a red liquid and used without further purification. ^1^H-NMR (CDCl3): δ 2.59 (bs, 2H), 1.47–1.32 (m, 16H), 1.12 (bs, 4H), 0.93 (t, ^3^*J*_HH_ = 7 Hz, 6H). ^13^C-NMR (CDCl_3_): δ 55.2, 34.8, 32.0, 26.2, 22.7, 14.1.

### 3.5. Synthesis of Homochiral Pentyl Cage

**Scheme 3:**
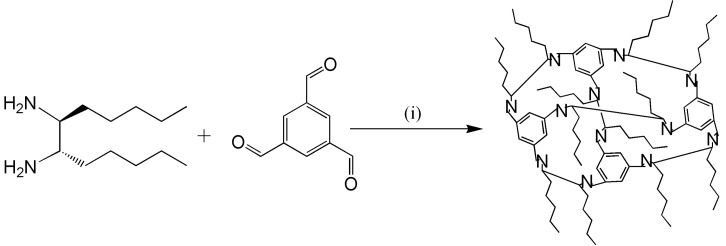
Synthesis of pentyl cage. *Reagents and Condition:* (i) TFA, CHCl_3_, 60 °C, 72 h.

The pentyl cage was synthesized according to a previous reference [37] (Scheme 3). Typically, a mixture of (*S*,*S*)-1,2-pentyl-1,2-diaminoethane (0.145 g, 0.73 mmol) dissolved in 3.3 mL of CHCl_3_ and 1,3,5-triformylbenzene (0.06 g, 0.40 mmol) dissolved in 2.3 mL CHCl_3_ was prepared, and trifluoroacetic acid (0.01 mL, 0.13 mmol) was added. The reaction mixture was stirred at 60 °C for 72 h. Then, the solvent was removed under reduced pressure and the crude purified by precipitation from acetone. Finally, crystals were grown by diffusing acetone in a solution of the pentyl cage in dichloromethane (0.060 g, yield 60%). ^1^H-NMR (CDCl_3_): δ 8.08 (s, 12H), 7.90 (s, 12H), 3.36–3.35 (d, ^3^*J*_HH_ = 8 Hz, 12H), 1.80–1.64 (m, 24H), 1.27–1.10 (m, 72H), 0.87 (t, ^3^*J*_HH_ = 6 Hz, 36H). ^13^C-NMR (CDCl_3_): δ 159.35, 136.62, 129.57, 75.41, 31.80, 26.10, 22.58, 14.12.

### 3.6. Capillary Pretreatment and Preparation of the Pentyl Cage-Coated Capillary Column

A fused-silica capillary column (15 m long × 0.25 mm i.d.) was pretreated according to the following method prior to coating: the column was firstly rinsed with 1.0 M NaOH for 3 h, deionized water for 1 h, 0.1 M HCl for 1 h and again using deionized water for a period of time to ensure the washing was neutral. Finally, the capillary was dried via a nitrogen purge for 6 h at 120 °C. Pentyl cage-coated capillary column was fabricated by a static method [34]. A mixture of a 1 mL solution of pentyl cage (3 mg∙mL^−1^) in dichloromethane and 1 mL solution of polysiloxane OV-1701 (4.5 mg∙mL^−1^) in dichloromethane was used to produce a pentyl cage-coated capillary column. The coating process was as follows: after the column was filled up with the stationary phase solution, one end of capillary column was sealed and the other end was connected to a vacuum system to gradually remove the solvent at 36 °C under vacuum to form a uniform film of the stationary phase on the inner surface of the capillary column. Finally, the pentyl cage-coated column was conditioned from 30 °C to 200 °C at a heating rate of 2 °C∙min^−1^ and held at 200 °C for 3 h under a flow of nitrogen.

## 4. Conclusions

A homochiral pentyl cage with a tetrahedral cage structure was obtained by an imine condensation reaction of (*S*,*S*)-1,2-pentyl-1,2-diaminoethane and 1,3,5-triformylbenzene. We have then fabricated a pentyl cage-coated capillary GC column via a static method. The pentyl cage-coated capillary column exhibited good selectivity and recognition ability for the GC separation of positional isomers and racemates. This results demonstrate that POCs-based chiral stationary phase are promising chiral selectors for enantioseparation in GC.

## Figures and Tables

**Table 1:** McReynolds constants of the pentyl cage-coated capillary column at 120 °C.

*X′*	*Y′*	*Z′*	*U′*	*S′*	Av.
37	185	102	203	126	130.6

*X′*, *Y′*, *Z′*, *U′* and *S′* refer to benzene, 1-butanol, 2-pentanone, 1-nitropropane, and pyridine, respectively.

**Table 2:** Separation factor (α) and resolution (Rs) for separation of positional isomers on the pentyl cage-coated capillary column.

Isomers	T (°C)	Separation Factor (α)		Resolution (Rs)	
		α_1_	α_2_	Rs_1_	Rs_2_
Dichlorobenzene	90	1.24	1.10	2.51	1.51
Dibromobenzene	110	1.23	1.12	2.83	1.57
Nitrochlorobenzene	115	1.14	1.67	2.91	9.59
Nitrobromobenzene	120	1.08	1.60	1.99	10.30

**Table 3:** Separation of racemates on the pentyl cage-coated capillary column.

Racemates	T (°C)	k_1_	α	Rs	*v* ^[a]^ (cm∙s^−1^)
Styrene oxide	110	3.24	1.07	1.59	10.8
*trans*-Stilbene oxide	178	5.76	1.15	4.94	11.9
1,2-Epoxybutane	50	3.15	1.10	0.86	11.0
1,2-Epoxyhexane	110	3.28	1.07	1.69	10.7
1-Bromo-2,3-epoxypropane	85	4.24	1.03	0.94	10.8
*n*-Butyl glycidyl ether	95	1.63	1.05	0.84	12.5
1-Methoxy-2-hydroxypropane	78	2.01	1.09	1.01	12.0
1-Methoxy-2-butanol	82	3.30	1.14	1.57	12.3
3-Butyn-2-ol	67	3.01	1.11	1.30	11.9
1-Phenylethanol	120	5.06	1.06	1.56	11.8
1-Phenyl-1-propanol	125	4.95	1.09	1.69	13.6
1-(4-Chlorophenyl)ethanol	135	5.89	1.03	1.18	12.3
2-Phenyl-1-propanol	130	5.77	1.03	0.57	13.0
1-Naphthylethanol	175	6.57	1.08	2.20	10.2
1-(2-Naphthyl)ethanol	175	6.50	1.03	0.58	11.4
Methyl 3-hydroxybutyrate	90	3.6	1.09	1.01	11.2
Ethyl 3-hydroxybutyrate	100	3.42	1.06	0.71	10.3
γ-Valerolactone	115	3.04	1.03	0.15	12.4

^[a]^ *v* is the linear velocity of the N_2_ carrier gas.

## Supplementary Materials

Supplementary materials can be accessed at: http://www.mdpi.com/1420-3049/21/11/1466/s1.

## Abbreviations

HPLC	high performance liquid chromatography
GC	gas chromatography
SFC	supercritical fluid chromatography
TLC	thin layer chromatography
CEC	capillary electrochromatography
MOFs	metal-organic frameworks
POFs	porous organic frameworks
POCs	porous organic cages
FID	flame ionization detector
SEM	scanning electron microscopy
TGA	thermogravimetric analysis
