# Supplementary material for: Application of Homochiral Alkylated Organic Cages as Chiral Stationary Phases for Molecular Separations by Capillary Gas Chromatography
Source: Molecules. 2016 Nov 8;21(11):1466. doi: 10.3390/molecules21111466 (PMC6274383; doi:10.3390/molecules21111466)
Supplement: Supplementary file 1 [file molecules-21-01466-s001.pdf]

Molecules **2016**, *21*, 1466; doi:10.3390/molecules21111466

Supplementary Materials: Application of Homochiral Alkylated Organic Cage as Chiral Stationary Phase for Molecular Separations by Capillary Gas Chromatography

Shengming Xie, Junhui Zhang, Nan Fu, Bangjin Wang, Cong Hu and Liming Yuan

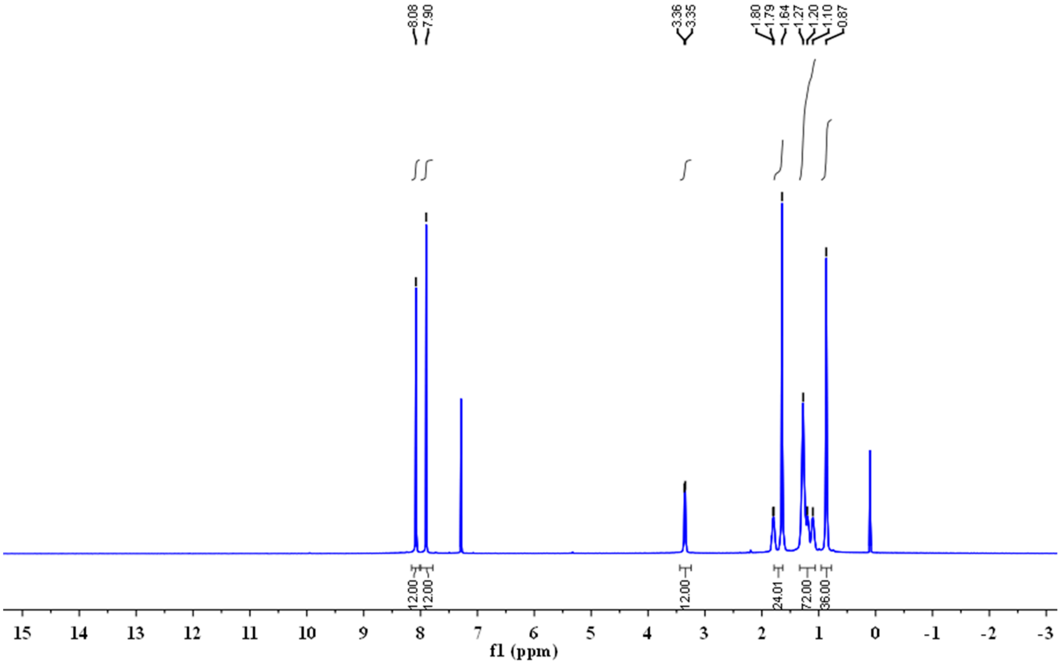

Figure S1. ^{1}H-NMR spectrum ($CDCl_3$) of the pentyl cage compound: δ 8.08 (s, 12H), 7.90 (s, 12H), 3.36–3.35 (d, $^3J_{HH}$ = 8 Hz, 12H), 1.80–1.64 (m, 24H), 1.27–1.10 (m, 72H), 0.87 (t, $^3J_{HH}$ = 6 Hz, 36H).

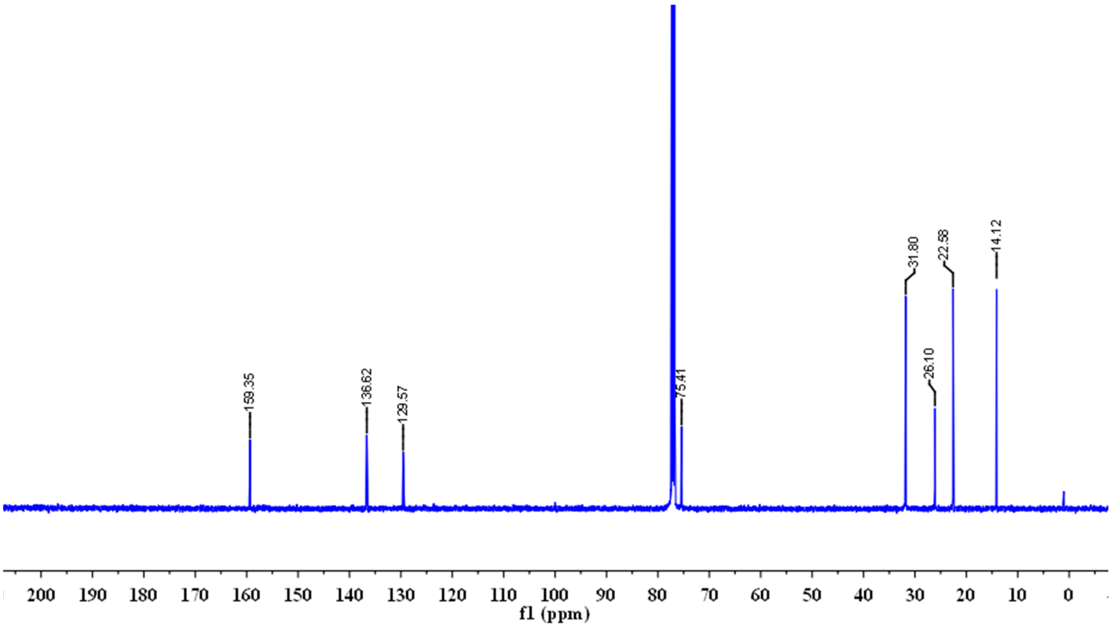

Figure. S2. ^{13}C-NMR spectrum ($CDCl_3$)of the pentyl cage compound: δ 159.35, 136.62, 129.57, 75.41, 31.80, 26.10, 22.58, 14.12.

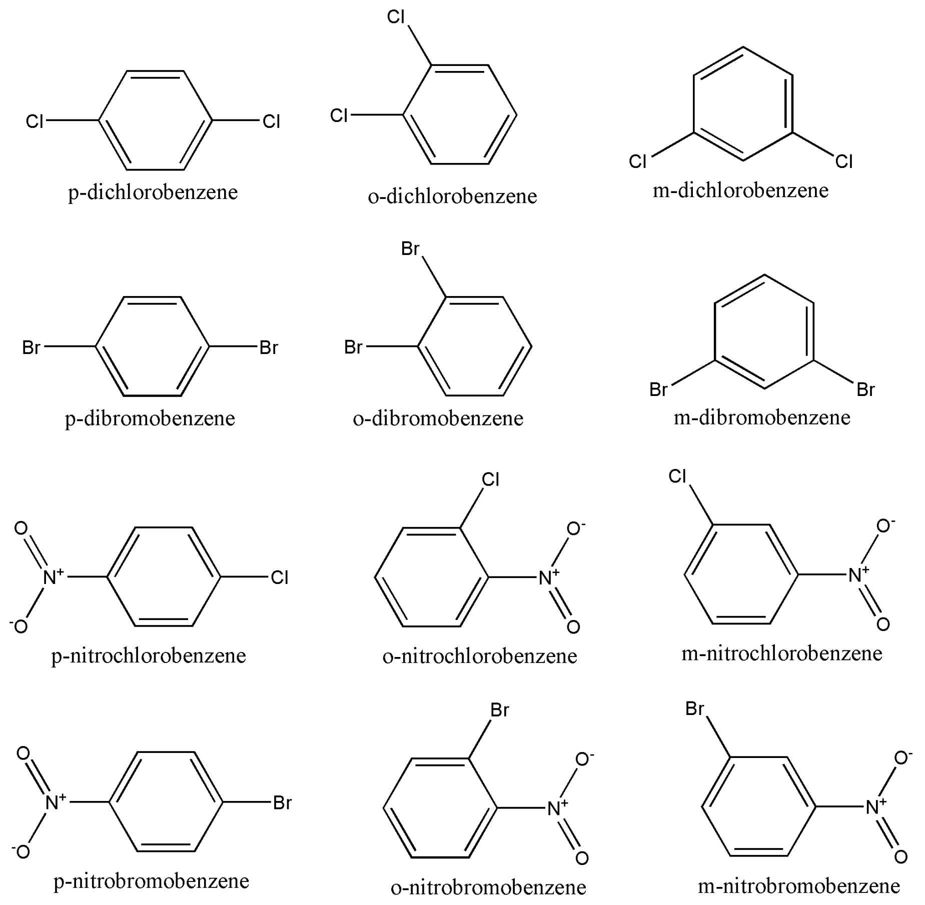

Figure S3. Structures of the positional isomers.

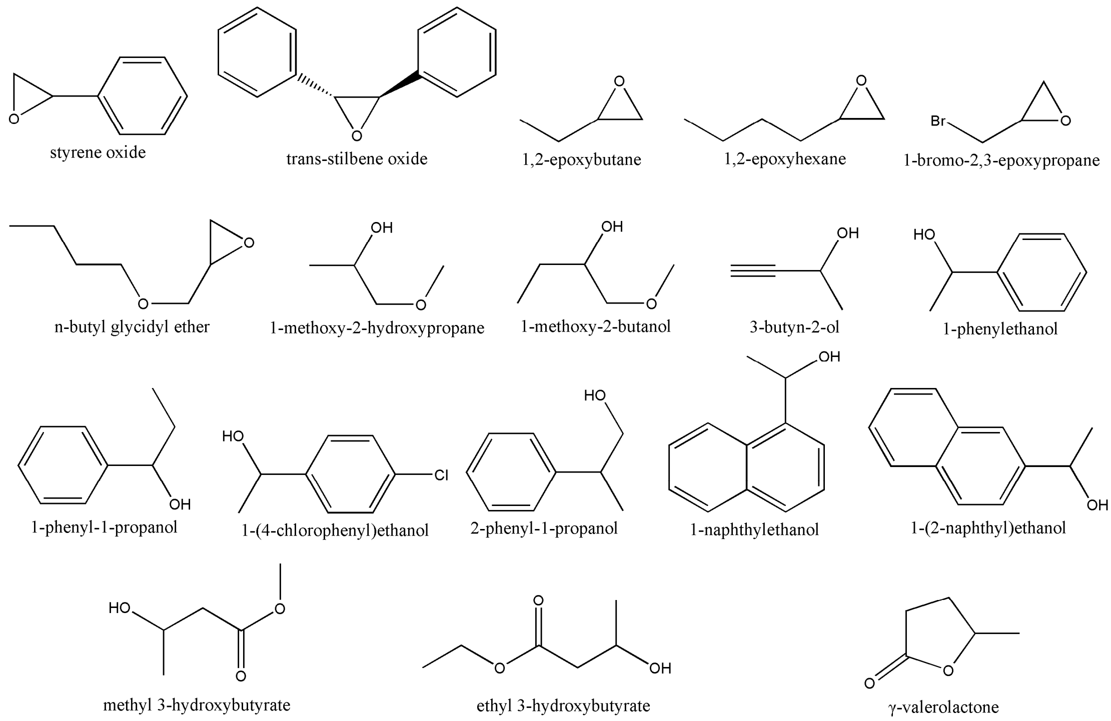

Figure S4. Structures of the racemates.

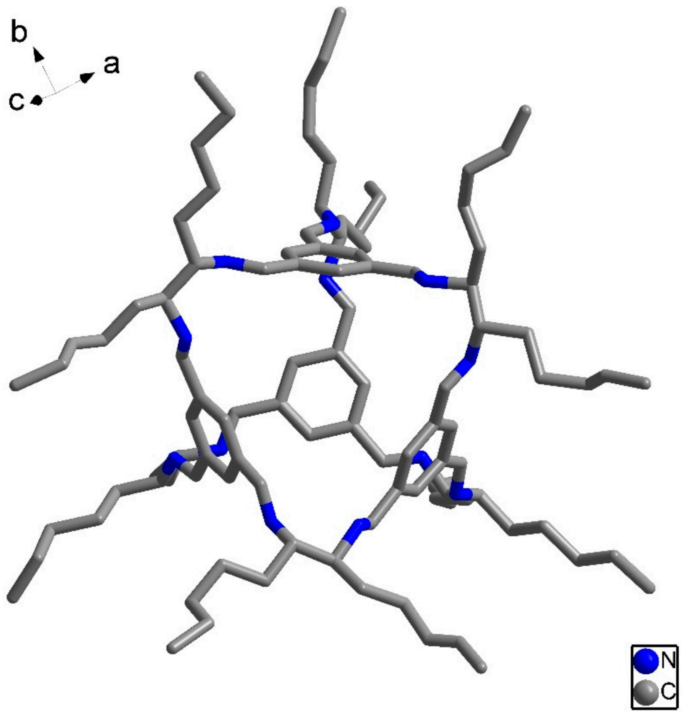

Figure S5. Structure of the pentyl cage with twelve *n*-pentyl tails. Hydrogens are omitted for clarity.
